# Platelet-Rich Plasma in Anterior Cruciate Ligament Reconstruction: An Updated Systematic Review and Meta-Analysis of Clinical and Radiological Outcomes

**DOI:** 10.3390/jcm15072526

**Published:** 2026-03-26

**Authors:** Amer Abdallah, Georges Assaf, Caroline Chahine, Ghadi Abou Orm, Sadek Jaber, Anthony Chalfoun, Julien Bou Chaaya, Hadi Soukarieh, Charbel Chaiban, Maher Ghandour, Ali Ghosn

**Affiliations:** 1 Department of Orthopaedic Surgery, Lebanese University, Beirut P.O. Box 6573/14, Lebanon; ameramd@gmail.com (A.A.); sadekjaber.md@gmail.com (S.J.); hadi.soukarieh@gmail.com (H.S.); 2 Department of Anesthesiology, Lebanese American University Medical Center, Beirut P.O. Box 13-5053, Lebanon; georges.assaf02@lau.edu.lb (G.A.); caroline.chahine@lau.edu.lb (C.C.); 3 Department of Orthopaedic Surgery, Lebanese American University Medical Center, Beirut P.O. Box 13-5053, Lebanon; ghadi.abouorm@lau.edu (G.A.O.); charbelchaiban97@gmail.com (C.C.); ali.ghosn.md@gmail.com (A.G.); 4 Department of Orthopaedic Surgery, Saint George Hospital University Medical Center, Beirut P.O.Box 166-378, Lebanon; anthony_chalfoun@hotmail.com (A.C.); julien.bouchaaya@gmail.com (J.B.C.)

**Keywords:** anterior cruciate ligament reconstruction, platelet-rich plasma, ligamentization, functional outcomes, knee stability

## Abstract

**Background/Objectives**: To evaluate the efficacy of platelet-rich plasma (PRP) as an adjunctive treatment in anterior cruciate ligament reconstruction (ACLR) and its impact on key clinical outcomes. **Methods**: A systematic search was conducted across five databases until 11 November 2024, including 33 randomized controlled trials (RCTs) that investigated PRP in ACLR. Outcomes analyzed included ligamentization (MRI hypointensity grades), pain VAS scores, functional scores (IKDC, Lysholm, Tegner), knee stability (KT-1000 arthrometer), and tunnel characteristics. Subgroup analyses were performed based on PRP application site, graft type, risk of bias, and follow-up duration. **Results**: PRP significantly enhanced ligamentization, particularly at 12 months, with marked reductions in MRI hypointensity grades. Patellar tendon grafts demonstrated the most substantial benefits. PRP also significantly reduced postoperative pain, with effects most pronounced in the early recovery period (1–9 months). However, the analgesic benefits diminished over time. Improvements in IKDC scores were observed only in studies with a high risk of bias, while Lysholm and Tegner scores showed no consistent differences between PRP and controls. Knee stability improved significantly with PRP, but this effect was limited to early follow-up periods (3 months). The heterogeneity in PRP preparation methods, application protocols, and patient populations limited the generalizability of the findings. **Conclusions**: PRP enhances ligamentization and provides short-term pain relief and stability benefits in ACLR. However, its impact on long-term functional recovery and other clinical outcomes remains limited and inconsistent. Standardization of PRP protocols and further high-quality research are necessary to refine its application and therapeutic potential.

## 1. Introduction

Anterior cruciate ligament (ACL) injuries represent a significant challenge in orthopedic and sports medicine due to their high prevalence and the long recovery periods they entail [1,2]. ACL reconstruction (ACLR) is widely recognized as the gold standard for restoring knee stability and function in active individuals, particularly athletes [3]. Despite advances in surgical techniques and postoperative rehabilitation protocols, a substantial proportion of patients fail to achieve optimal outcomes, including a full return to pre-injury activity levels and long-term joint health [4,5]. Persistent issues such as graft maturation, integration, and postoperative pain remain critical barriers to recovery [6,7].

Ligamentization—the biological remodeling of graft tissue into ligament-like structures—plays a pivotal role in the success of ACLR [8]. This process encompasses several stages, including necrosis, cellular infiltration, collagen synthesis, and revascularization. While this natural progression is essential for graft incorporation, it is inherently slow and subject to variability influenced by patient factors, graft type, and surgical technique [9]. Enhancing this process has therefore become a key focus of research, with the aim of improving clinical outcomes and reducing recovery times.

Platelet-rich plasma (PRP) has emerged as a promising biological adjunct in ACLR [10]. Derived from autologous blood, PRP is enriched with growth factors such as platelet-derived growth factor (PDGF), transforming growth factor-beta (TGF-β), and vascular endothelial growth factor (VEGF), which are known to facilitate tissue repair and regeneration [11]. Preclinical and early clinical studies have suggested that PRP may enhance graft maturation, promote bone–tunnel healing, and modulate inflammatory responses [12,13]. However, the evidence remains inconsistent, with conflicting findings regarding its efficacy in improving ligamentization, functional outcomes, and pain relief [14,15].

The clinical application of PRP in ACLR is further complicated by variations in its preparation, dosing, and administration. Differences in patient populations, graft types, and outcome measures have contributed to the heterogeneity of results observed across studies, limiting the generalizability of findings and precluding definitive clinical recommendations [15,16]. Consequently, there is a pressing need for a systematic evaluation of the available evidence to better understand PRP’s role in ACLR and its potential to address the unmet challenges of graft healing and functional recovery.

A major challenge in interpreting PRP-related outcomes is the lack of standardized biological characterization. PRP is not a uniform product; its cellular composition, platelet concentration, leukocyte content, activation method, injected volume, and total platelet dose vary considerably depending on preparation protocols. Contemporary classification systems, such as the PAW and MARSPILL classifications, emphasize that the total platelet dose and biological composition may represent critical determinants of clinical efficacy. However, many clinical trials fail to report sufficient details regarding the platelet concentration or dosing, limiting reproducibility and cross-study comparisons. This heterogeneity complicates the interpretation of PRP efficacy in ACLR and may partially explain inconsistent findings across trials.

This study aims to systematically review and synthesize the evidence on the efficacy of PRP in ACLR, focusing on its impact on ligamentization, pain, functional scores, knee stability, and other clinically relevant outcomes. By integrating data from randomized controlled trials and leveraging advanced meta-analytic techniques, this research seeks to provide a comprehensive and nuanced understanding of PRP’s therapeutic potential.

## 2. Materials and Methods

### 2.1. Design and Literature Search

This systematic review and meta-analysis was conducted in line with the recent PRISMA [17] and AMSTAR [18] guidelines. The study protocol was not registered on PROSPERO given the presence of an already registered protocol at the time of registration (CRD420251142319). The PRISMA checklist is provided as Supplementary File S1.

We searched PubMed, Scopus, Web of Science, Cochrane Library, and Google Scholar (first 200 records) [19] up to 11 November 2024. The search strategy included the following keywords: “anterior cruciate ligament”, platelet, and random*. The full search query can be found in Table S1. Citations were filtered based on their titles and abstracts. No restrictions were applied regarding the original language of publication. Manual searches included reviewing reference lists and related articles on PubMed [20] as well as on Google Software.

### 2.2. Selection Strategy

The study selection was carried out following the PICOS (Population, Intervention, Comparison, Outcomes, and Study Design) theme [21].

The inclusion criteria were as follows:

- Population: Patients who underwent ACL reconstruction.
- Intervention: PRP.
- Comparison: Control.
- Outcome: Pain (as primary endpoint) and clinical/radiological measures (secondary endpoints).
- Study Design: Only randomized controlled trials (RCTs) were considered.

The exclusion criteria included the following:

- Non-original research.
- Non-randomized studies of intervention.
- Abstract-only publications.
- Case reports and case series.
- Case–control and cohort studies.
- Duplicate studies or studies with overlapping datasets (as in post hoc studies or subgroup analytic studies).
- Meniscal reconstruction (not ACL reconstruction).
- Animal studies plus in vivo or in vitro studies.

### 2.3. Data Collection and Outcomes

The data extraction sheet was formatted using Microsoft Excel, which is made up of four parts. The first part included citation-specific data (last name of first author, year of publication and investigation, country, study design, and total sample). The second part was related to patients’ characteristics (i.e., age, gender, graft type, intervention groups, and follow-up). When available, data regarding PRP preparation protocol, platelet concentration, and reported biological characteristics were extracted. Total platelet dose could not be calculated because most trials did not report both baseline platelet counts and final PRP concentrations. Consequently, formal subclassification of PRP formulations (e.g., PAW or MARSPILL systems) was not feasible.

For the purposes of this review, PRP was defined broadly as any autologous platelet-derived concentrate applied locally during ACL reconstruction with the intention of enhancing graft healing. This included products labeled as platelet-rich plasma (PRP), plasma rich in growth factors (PRGFs), platelet-rich fibrin (PRF), autologous platelet concentrate (APC), and platelet concentrate (PC). Because detailed biological characterization (e.g., leukocyte content, fibrin architecture, platelet concentration, activation method, total platelet dose) was inconsistently reported across trials, subclassification into biologically distinct categories was not feasible.

The third part included the outcome data. The primary outcome was postoperative pain, measured by the visual analogue scale (VAS). Secondary endpoints included clinical and radiological measures. Clinical measures included Lysholm score, Tegner score, IKDC score, and knee intensity (measured by KT-1000 arthrometry), while radiological measures included tibial/femoral tunnel diameter, ligamentization (MRI hypointensity grades 1–4: mild–diffuse), and graft maturation, as measured by the signal noise quotient (SNQ). The fourth part included the methodological quality assessment part.

### 2.4. Risk-of-Bias Assessment

For all included RCTs, the revised version of the Cochrane’s risk-of-bias tool was used. The methodological quality was assessed over five domains: randomization, deviation from intended interventions, missing outcome data, measurement bias, and selective reporting. Overall, a study was deemed as high risk, low risk, or some concerns.

### 2.5. Statistical Analysis

All statistical analyses were conducted using STATA version 18 (StataCorp, USA). Because the studies included in the quantitative synthesis were highly heterogeneous, a random-effects model was applied, with pooled estimates calculated using the restricted maximum likelihood (REML) method to reduce potential bias related to incomplete data [22]. Owing to substantial variability in outcome reporting—such as differences in follow-up intervals, graft types, and injection sites—deriving a single overall pooled estimate was considered inappropriate. Accordingly, subgroup analyses were performed based on follow-up duration, graft type, PRP injection site, and risk of bias.

Heterogeneity was quantified using the I^2^ statistic, with significant heterogeneity defined as I^2^ > 40% [23]. The robustness of the findings was further examined through sensitivity analyses, in which Galbraith plots were used to detect potential outliers. Publication bias was assessed using funnel plots and tests for asymmetry [24]. These analyses showed that the results remained stable after sensitivity testing, and no significant evidence of publication bias was detected.

## 3. Results

### 3.1. Literature Search Results

**Figure 1:**
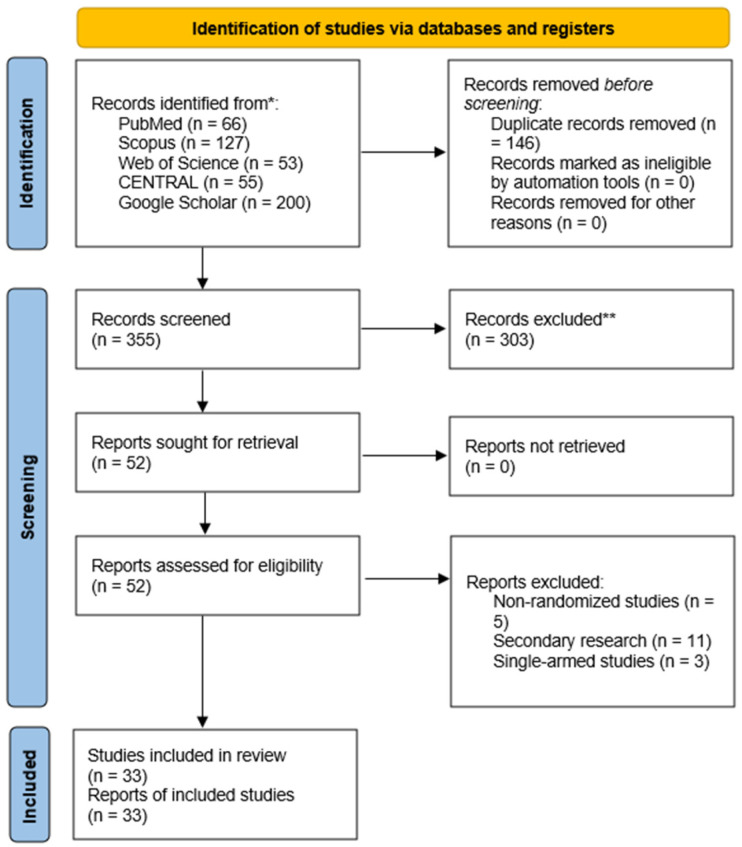
A PRISMA flow diagram showing the results of the literature search. ** the number of citations before duplicate removal; * the number of citations after title/abstract screening and duplicate removal.

The systematic search across multiple databases yielded 501 records (Figure 1). After removing 146 duplicates, 355 unique articles remained for screening. During the title and abstract review, 303 records were excluded. Subsequently, 52 full-text articles were retrieved and assessed for eligibility, all of which were successfully obtained. Among these, 19 studies were excluded for the following reasons: non-randomized study design (*n* = 5), secondary research articles (*n* = 11), and single-arm studies (*n* = 3). Ultimately, 33 randomized controlled trials (RCTs) fulfilled the eligibility criteria and were included in the meta-analysis [25,26,27,28,29,30,31,32,33,34,35,36,37,38,39,40,41,42,43,44,45,46,47,48,49,50,51,52,53,54,55,56,57].

### 3.2. Baseline Characteristics of Included Studies

The baseline clinicodemographic characteristics of the included studies and examined patients are presented in Table 1. Most evidence came from Spain (6 RCTs, 17.65%) and China (4 RCTs, 11.76%). A total of 1913 patients who underwent ACL reconstruction were allocated to either the PRP (981 patients) or control group (932 patients). The follow-up ranged from as short as 1 day to 24 months. The types of grafts used in ACL reconstruction included hamstring tendon (18 RCTs), patellar tendon (9 RCTs), peroneus longus tendon (1 RCT), quadriceps tendon (1 RCT), and semitendinosus tendon (1 RCT). A description of patients’ age and gender is provided in Table 1.

The included RCTs utilized various platelet-derived formulations, including platelet-rich plasma (PRP), plasma rich in growth factors (PRGFs), platelet-rich fibrin (PRF), autologous platelet concentrate (APC), and platelet concentrate (PC). Terminology and preparation protocols varied considerably across studies. Detailed reporting of leukocyte content, red blood cell contamination, fibrin matrix characteristics, and activation protocols was inconsistent. Due to insufficient biological characterization and limited numbers within each product category, separate quantitative pooling by product type was not statistically feasible.

### 3.3. Risk-of-Bias Assessment

**Figure 2:**
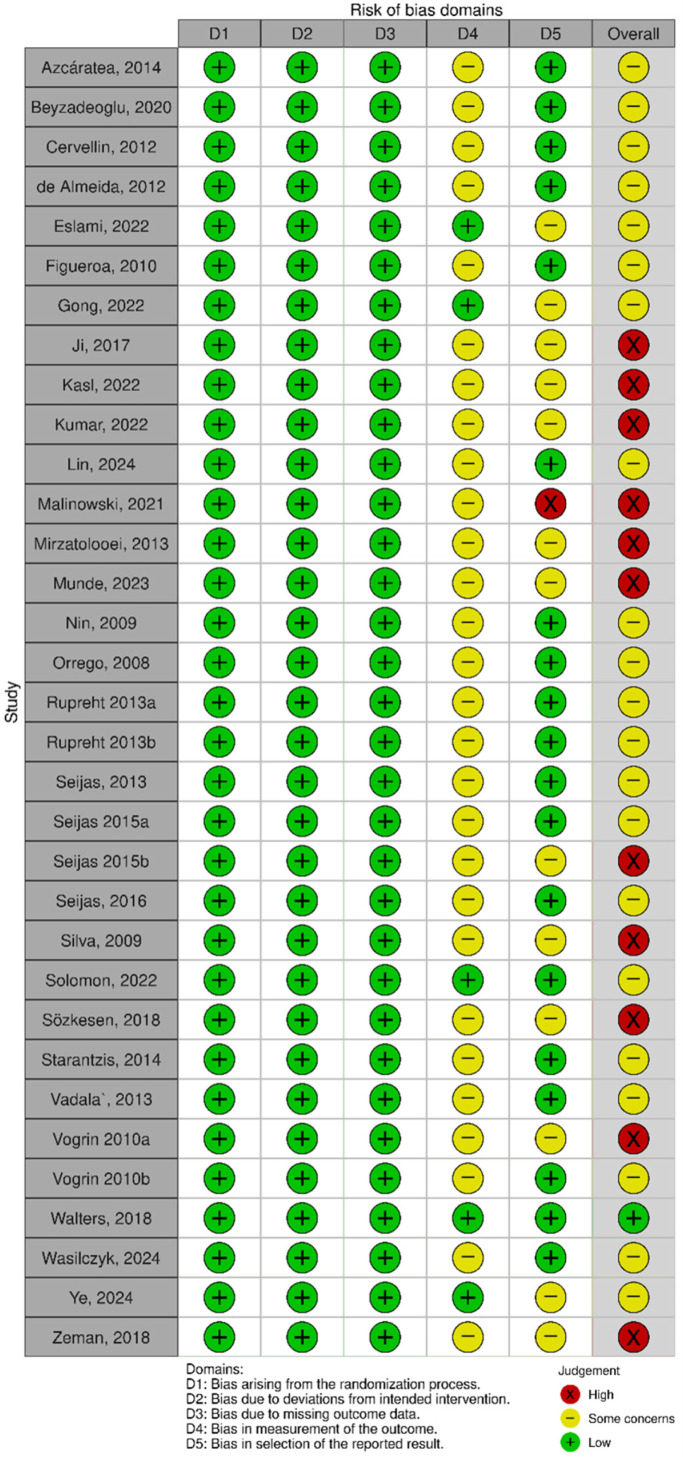
A summary of the risk of bias of included randomized controlled trials comparing PRP to control in patients undergoing ACL reconstruction [25,26,27,28,29,30,31,32,33,34,35,36,37,38,39,40,41,42,43,44,45,46,47,48,49,50,51,52,53,54,55,56,57].

The overall risk-of-bias assessment is summarized in Figure 2. Among the 33 included RCTs, most were judged as having some concerns (21/33, 63.6%), while 11 (33.3%) were at high risk of bias. Only one study (3.0%) was considered low risk.

### 3.4. Primary Endpoint (VAS Score)

**Figure 3:**
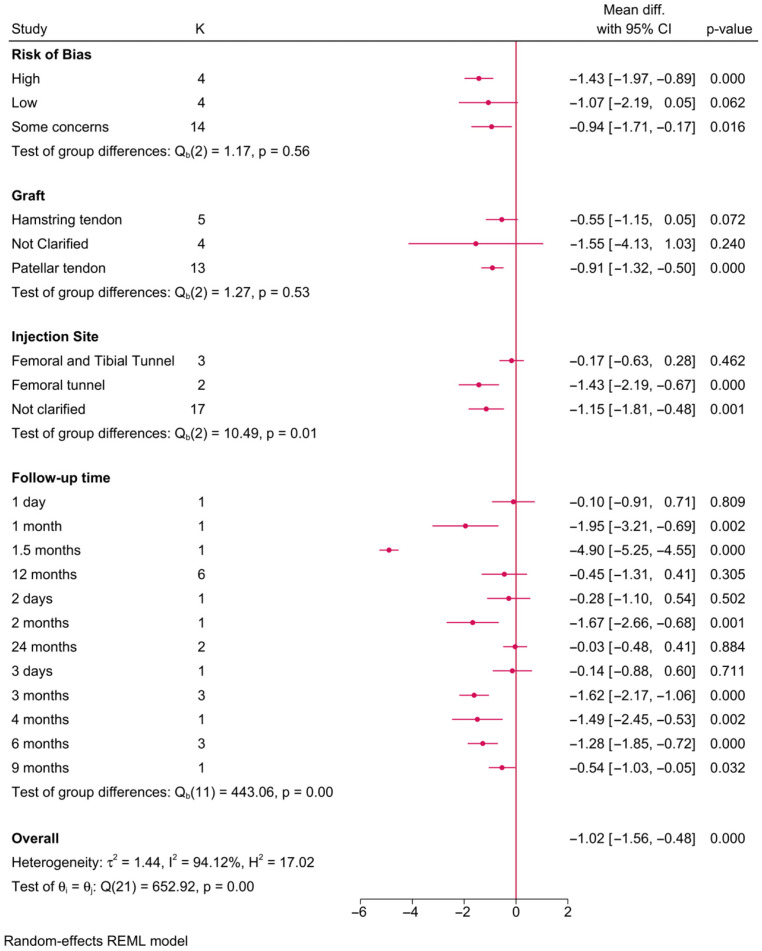
A forest plot showing the difference in postoperative pain score between PRP and control stratified by risk-of-bias level, graft type, injection site, and follow-up time. The *p*-values displayed represent subgroup interaction tests evaluating whether effect sizes differ across categories. I^2^ values indicate the degree of statistical heterogeneity among included studies within each subgroup.

Twenty-two RCTs investigated the impact of PRP on pain scores (Figure 3). The meta-analysis showed that the injection site (*p* = 0.01) and follow-up time (*p* = 0.001) had a significant effect modification on postoperative pain. For instance, in studies where PRP was injected into the femoral tunnel, a significant reduction in pain score was observed compared to the control (MD = −1.43; 95% CI: −2.19; −0.67]. However, no change was observed with PRP injected in both the femoral and tibial tunnels simultaneously. Additionally, the observed reduction in pain score in favor of PRP was observed from the first month (MD = −1.95; 95% CI: −3.21, −0.69] till the ninth month [MD = −0.54; 95% CI: −1.03, −0.05] of follow-up. However, this difference was diminishing over time.

Of note, the reduction in pain score in favor of PRP was observed only in RCTs with a high risk of bias or those with some concerns, while RCTs of a low risk of bias exhibited no significant difference between both groups (MD = −1.07; 95% CI: −2.19, 0.05]. Although differences in pain were observed with different graft types, this factor did not play a significant effect-modifying role on postoperative pain (*p* = 0.53). That being said, the reduction in pain scores was noted only with the use of the patellar tendon graft (MD = −0.91; 95% CI: −1.32, −0.50), while the hamstring tendons graft showed no difference in postoperative pain.

### 3.5. Secondary Endpoints

#### 3.5.1. IKDC Score

**Figure 4:**
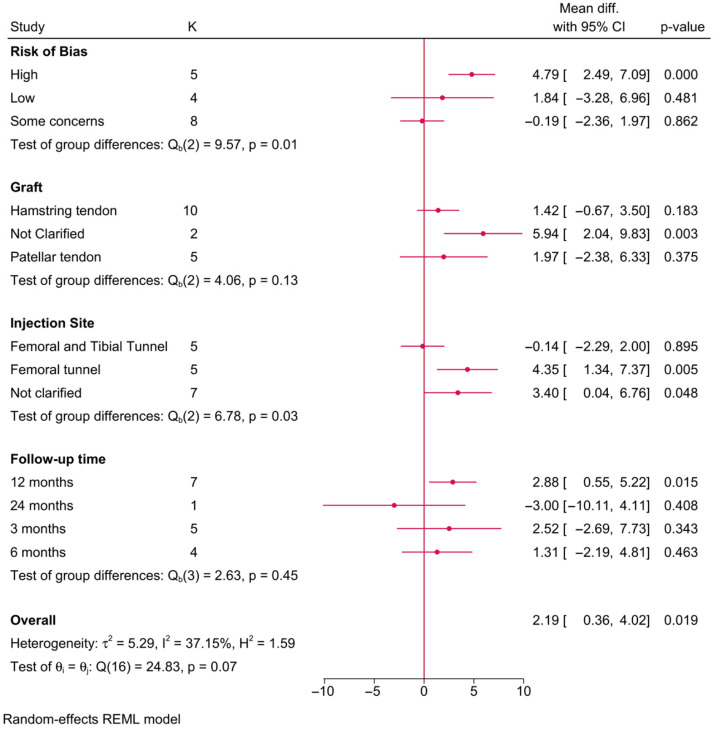
A forest plot showing the difference in postoperative IKDC score between PRP and control stratified by risk-of-bias level, graft type, injection site, and follow-up time.

Seventeen RCTs investigated the impact of PRP on postoperative IKDC scores (Figure 4). The risk of bias (*p* = 0.01) and injection site (*p* = 0.03) exhibited a significant effect-modifying role on the postoperative IKDC score. For instance, a significant increase in the postoperative IKDC score in favor of PRP was only observed in RCTs of a high risk of bias (MD = 4.79; 95% CI: 2.49, 7.09), while those with a low risk or some concerns exhibited no difference. Additionally, this increase in the IKDC score of PRP over the control group was observed in those who received PRP injection into the femoral tunnel alone (MD = 4.35; 95%CI: 1.34, 7.37), while those injected into both the femoral and tibial tunnels simultaneously exhibited no difference. Noteworthy, the observed difference in the IKDC score between PRP and the control was only observed at 12 months of follow-up (MD = 2.88; 95% CI: 0.55, 5.22) and not before that time (3–6 months).

#### 3.5.2. Lysholm Score

Sixteen RCTs investigated the impact of PRP on postoperative Lysholm scores. None of the investigated variables showed a significant effect-modifying role on the postoperative Lysholm score, including the risk of bias (*p* = 0.14), graft type (*p* = 0.82), PRP injection site (*p* = 0.77), or follow-up time (*p* = 0.63) (Supplementary Figure S1). No significant difference in the postoperative Lysholm score was observed between the PRP and control groups across all investigated subgroups/comparisons.

#### 3.5.3. Tegner Score

Six RCTs investigated the impact of PRP on postoperative Tegner scores. None of the investigated variables showed a significant effect-modifying role on the postoperative Tegner score, including the risk of bias (*p* = 0.30), graft type (*p* = 0.72), PRP injection site (*p* = 0.72), or follow-up time (*p* = 0.25) (Supplementary Figure S2). No significant difference in the postoperative Tegner score was observed between the PRP and control groups across all investigated subgroups/comparisons.

#### 3.5.4. Graft Maturation (SNQ)

Two RCTs investigated the impact of PRP on graft maturation as measured by the SNQ (Supplementary Figure S3). None of the examined variables showed a significant effect-modifying role on the SNQ. A significant reduction in the postoperative SNQ was observed in favor of PRP compared to the control only in those who had a hamstring tendon graft (MD = −0.63; 95% CI: −0.99, −0.27) and at 12 months of follow-up.

#### 3.5.5. Tunnel Diameters (mm)

**Figure 5:**
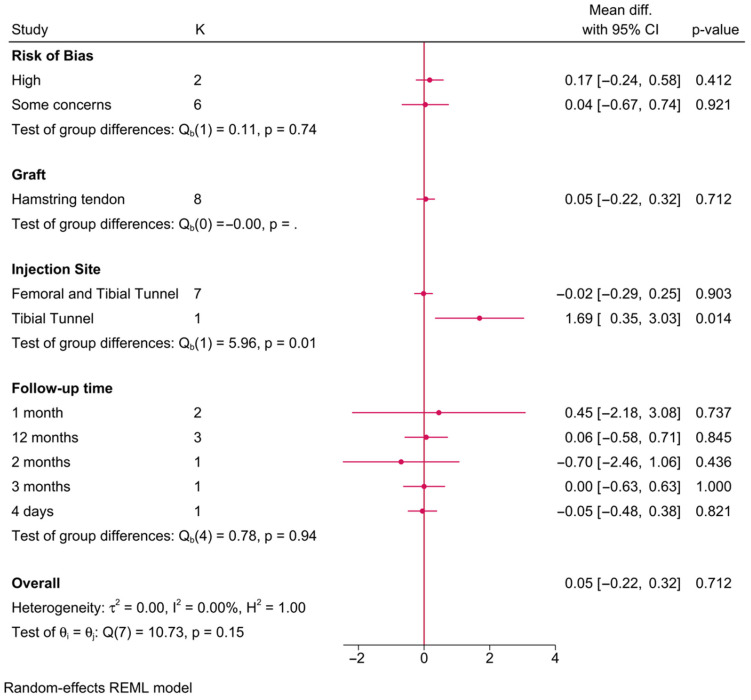
A forest plot showing the difference in postoperative tibial tunnel diameter between PRP and control stratified by risk-of-bias level, graft type, injection site, and follow-up time. Subgroup *p*-values reflect interaction testing between categories, while I^2^ values quantify the percentage of variability attributable to between-study heterogeneity rather than sampling error.

Eight RCTs investigated the association between PRP and tibial tunnel diameter (Figure 5). The injection site was the only significant effect modifier of the tibial tunnel diameter (*p* = 0.01). PRP showed a significant increase in tibial tunnel diameter compared to the control group, only where PRP was injected into the tibial tunnel alone (MD = 1.69; 95% CI: 0.35, 3.03), with no change when injected into both the femoral and tibial tunnels simultaneously.

Seven RCTs investigated the association between PRP and femoral tunnel diameter (Supplementary Figure S4). Risk of bias was barely a significant effect modifier of the effect of PRP on the postoperative femoral tunnel diameter (*p* = 0.05). That being said, no significant difference between PRP and the control was observed across all subgroups.

#### 3.5.6. Knee Stability (KT-1000 Arthrometric Measure—mm)

**Figure 6:**
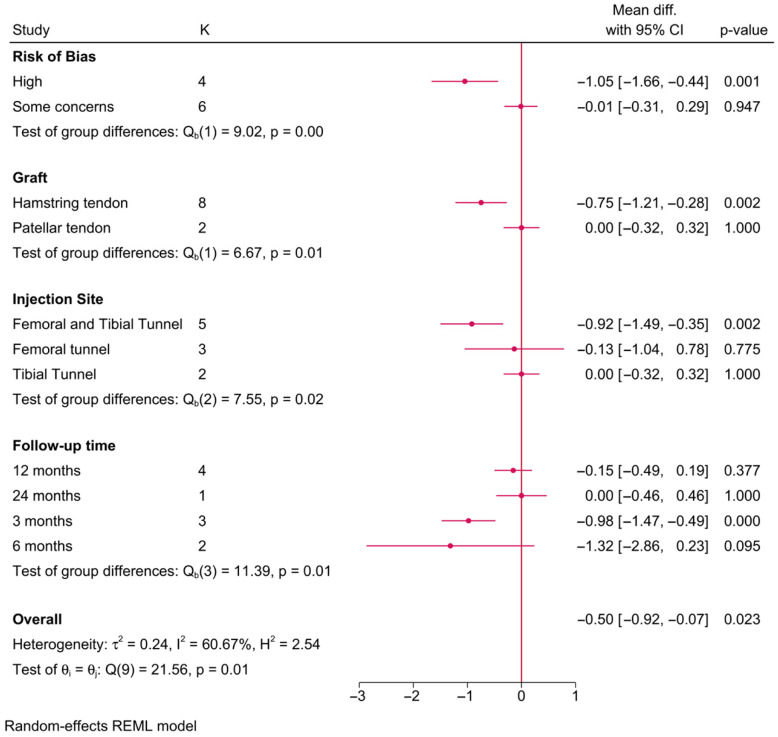
A forest plot showing the difference in postoperative knee stability (KT-1000 score) between PRP and control stratified by risk-of-bias level, graft type, injection site, and follow-up time.

Ten RCTs investigated the impact of PRP on knee stability, as measured by the KT-1000 arthrometric measure (Figure 6). The risk of bias (*p* = 0.001), graft type (*p* = 0.01), injection site (*p* = 0.02), and follow-up time (*p* = 0.01) significantly modified the effect of PRP on postoperative knee stability. For instance, PRP was associated with a significantly lower KT-1000 score compared to the control; however, this difference was only observed in RCTs with a high risk of bias (MD = −1.05; 95% CI: −1.66, −0.44), surgeries utilizing hamstring tendon grafts (MD = −0.75; 95% CI: −1.21, −0.28), and when PRP was injected into both the femoral and tibial tunnels simultaneously (MD = −0.92; 95% CI: −1.49, −0.35). This effect was observable only at 3 months of follow-up (MD = −0.98; 95% CI: −1.47, −0.49) and not beyond or before that time point.

#### 3.5.7. Ligamentization (MRI Hypointensity Grade)

PRP had a significant impact on promoting ligamentization and enhancing graft maturation (Table 2). Specifically, PRP was associated with a marked reduction in the odds of achieving higher grades of hypointensity, which correspond to less mature grafts. The odds ratio (OR) for achieving Grade 4 (diffusely hypointense) outcomes was notably reduced in the PRP group compared to the controls, suggesting a positive role of PRP in accelerating graft integration and maturation.

Subgroup analyses provided additional insights. Studies utilizing patellar tendon grafts demonstrated a significant improvement in ligamentization outcomes in the PRP group, with an OR of 2.59 (95% CI: 1.21–5.52) for achieving better MRI hypointensity grades compared to the controls. In contrast, outcomes for hamstring tendon grafts were less conclusive, highlighting a potential graft-specific effect of PRP. Further, when PRP was injected into the femoral and tibial tunnels, the results consistently showed improved ligamentization metrics.

Temporal analysis revealed that the benefits of PRP on ligamentization were more pronounced at longer follow-up durations, particularly at 12 months. This suggests that PRP may exert its greatest effect during the later stages of graft remodeling. However, variability in the findings was observed across studies, influenced by factors such as the risk of bias, follow-up time, and PRP preparation and application protocols.

## 4. Discussion

The findings of our systematic review and meta-analysis underscore the complex and heterogeneous nature of the impact of PRP on ACLR. The analysis revealed nuanced insights into the role of PRP in ligamentization, graft maturation, and other clinical outcomes, while highlighting persistent gaps in the literature. Here, we contextualize these findings within the broader landscape of ACLR research.

### 4.1. Pain Reduction

PRP demonstrated a significant, albeit transient, reduction in postoperative pain. This effect was most pronounced in studies with femoral tunnel injections and at earlier follow-up points (1 to 9 months). However, the magnitude of pain reduction diminished over time, suggesting that PRP primarily accelerates early healing rather than providing sustained analgesic effects. This aligns with the hypothesized mechanisms of PRP, wherein growth factors like platelet-derived growth factor and vascular endothelial growth factor attenuate early inflammatory responses and promote tissue repair [11,15]. Notably, the association between PRP and pain reduction was predominantly observed in studies with a high or moderate risk of bias, raising questions about its clinical reliability [14,16].

### 4.2. Functional Scores (Lysholm, Tegner, and IKDC)

The impact of PRP on functional scores revealed a complex picture. While PRP significantly improved IKDC scores at 12 months of follow-up, these effects were limited to studies with a higher risk of bias and injections targeting femoral tunnels. Similarly, the Lysholm and Tegner scores showed no meaningful differences between the PRP and control groups across most studies. These findings suggest that PRP’s functional benefits, if present, may be context-dependent, influenced by factors such as the graft type, injection site, and study design [16,58]. This is consistent with prior meta-analyses, which have highlighted PRP’s limited role in enhancing patient-reported outcomes [12,59].

### 4.3. Knee Stability (KT-1000)

Knee stability, as measured by KT-1000 arthrometry, showed significant improvement with PRP in studies that utilized hamstring tendon grafts and employed dual-site injections (femoral and tibial tunnels). However, these effects were only evident at the 3-month follow-up and were not sustained over time. This transient improvement in stability may reflect PRP’s early modulation of graft integration, but the lack of long-term benefits raises concerns about its durability [16,59].

### 4.4. PRP and Ligamentization

Our analysis demonstrated that PRP was significantly associated with enhanced ligamentization as measured by MRI hypointensity grades, particularly at later stages of follow-up (12 months). This effect was most pronounced when PRP was used in conjunction with patellar tendon grafts, which may be attributable to the unique biological environment created by these grafts. The odds ratios favoring PRP for improved ligamentization outcomes corroborate earlier reports suggesting that growth factors within PRP, such as platelet-derived growth factor and transforming growth factor-beta, enhance fibroblast activity, angiogenesis, and collagen synthesis [11,12].

Despite these promising results, the effect of PRP on ligamentization varied based on the injection site and follow-up duration. Studies where PRP was administered to the femoral and tibial tunnels reported more consistent benefits, aligning with preclinical evidence that PRP enhances graft–bone integration [58,59]. However, variability in PRP preparation, application techniques, and patient populations across studies likely contributes to inconsistent results, emphasizing the need for standardized protocols [15,16].

A notable divergence in the literature pertains to the impact of PRP on clinical outcomes such as pain and functional scores. While our study observed significant pain reduction in the early postoperative period, particularly with injections targeting specific sites, the benefits diminished over time. These findings parallel prior reviews suggesting that PRP may accelerate early recovery but fails to provide sustained long-term benefits [16,59].

### 4.5. Temporal Pattern of Effects and Heterogeneity

An important observation across outcomes was the predominantly early and time-dependent nature of PRP-associated benefits. Reductions in postoperative pain were most evident within the first 1–9 months, while improvements in knee stability were primarily observed at 3 months and were not sustained thereafter. Several explanations may account for this pattern. Firstly, PRP-derived growth factors such as PDGF and TGF-β are thought to exert their greatest influence during the early inflammatory and proliferative phases of graft healing. During this period, enhanced angiogenesis, cellular infiltration, and collagen synthesis may accelerate initial graft integration. However, as graft remodeling progresses into the maturation phase, the biological contribution of exogenous platelet-derived factors may diminish, leading to convergence between the PRP and control groups over time. Secondly, standardized postoperative rehabilitation protocols may reduce early between-group differences as mechanical loading and neuromuscular adaptation become dominant determinants of functional recovery. Thus, any biologically mediated acceleration in early healing may not necessarily translate into sustained clinical superiority. Thirdly, patient-reported outcome measures such as IKDC, Lysholm, and Tegner scores may exhibit ceiling effects at later follow-up intervals, limiting their sensitivity to detect subtle long-term differences. Finally, heterogeneity in patient populations, graft types, and PRP preparation methods likely contributed to the variability in early responses. While certain subgroups (e.g., specific graft types or injection sites) demonstrated transient benefits, these effects were not consistently reproducible across broader populations.

### 4.6. Clinical Implications

The clinical relevance of PRP in ACL reconstruction should be interpreted cautiously. Although PRP was associated with improved ligamentization and reductions in postoperative pain, these effects were largely time-dependent and most evident during early follow-up. Similarly, improvements in knee stability (KT-1000 measurements) were observed primarily at 3 months and were not sustained at later time points. Moreover, several favorable findings were restricted to specific subgroups or studies with a higher risk of bias.

Importantly, consistent long-term improvements in functional outcomes such as Lysholm, Tegner, and IKDC scores were not demonstrated across the broader evidence base. Therefore, current data do not support the routine use of PRP as a standard adjunct in ACL reconstruction.

PRP may represent a biologically plausible adjunct with potential short-term benefits in selected contexts; however, its role in enhancing durable clinical recovery remains uncertain. Future high-quality randomized trials with standardized PRP characterization and extended follow-up are necessary before definitive clinical recommendations can be made.

### 4.7. Limitations and Future Directions

Several limitations must be acknowledged. The heterogeneity in the trials’ risk-of-bias degree, PRP preparation methods, and outcome assessments complicates the synthesis of evidence. Additionally, the predominance of studies with a high risk of bias underscores the need for rigorous randomized controlled trials with standardized methodologies. Another important limitation relates to pooling across biologically heterogeneous platelet-derived products. Although labeled differently (PRP, PRGF, PRF, APC, PC), these products were consistently applied as autologous platelet-enriched adjuncts during ACL reconstruction. However, the preparation techniques, fibrin structure, leukocyte content, and activation status likely differed across trials. Because most studies did not report sufficient laboratory characterization, biologically stratified meta-analysis was not possible.

A critical limitation of the included literature is the absence of standardized PRP characterization. Contemporary orthobiologic concepts emphasize that the total platelet dose, leukocyte content, activation status, and preparation protocol may represent key determinants of therapeutic efficacy. However, most RCTs failed to report the platelet concentration relative to the baseline, leukocyte content, or injected platelet mass. Without these parameters, formal classification using systems such as PAW or MARSPILL was not feasible. The inability to account for biological variability likely contributed to the heterogeneity and may partially explain the inconsistent clinical findings observed across studies.

Another limitation relates to the limited availability of direct mechanistic data within the included trials. Most randomized studies focused on clinical, radiological, and functional outcomes without incorporating histological analysis, quantitative vascular assessment, biomarker evaluation, or standardized biological profiling of PRP formulations. Consequently, interpretations regarding the underlying biological mechanisms of PRP in ligamentization and graft maturation remain indirect and largely extrapolated from preclinical research. This limits the ability to establish definitive causal pathways between PRP application and observed clinical effects.

Future research should focus on elucidating the mechanisms underlying PRP’s variable efficacy, optimizing its preparation and application, and identifying patient subgroups most likely to benefit. Comparative trials investigating different PRP formulations and dosing regimens could further refine its clinical utility.

## 5. Conclusions

PRP holds promise as an adjunctive therapy in ACLR, particularly for enhancing ligamentization and early postoperative recovery. However, its role in improving long-term functional outcomes remains limited and contentious. Standardization of PRP preparation protocols, including systematic reporting of the platelet concentration, leukocyte composition, activation methods, injected volume, and total platelet dose, is essential to enable reproducibility and clarify its therapeutic role in ACLR. Our findings provide a foundation for future investigations aimed at bridging the gap between experimental promise and clinical applicability in ACLR.

## Figures and Tables

**Table 1:** Baseline characteristics of randomized controlled trials comparing PRP to control in ACL reconstruction.

Author (YOP)	Country	Design	Registration	Graft	YOI	Group	PRP Injection Site	Sample Size	Age		**Gender**		**Follow-Up**
									Mean	SD	Male	Female	
**Mirzatolooei (2013)** [37]	Iran	RCT	NR	Hamstring tendon	February 2011–February 2012	PRP	Femoral and Tibial Tunnel	25	26.4	(18–40)	20	3	3 months
						Control	-	25	26.9	(18–40)	22	1	
**de Almeida (2012)** [28]	Brazil	RCT	NR	Patellar tendon	-	PRP	NR	12	25.8	(18–44)	10	2	6 months
						Control	-	15	23.1	(15–34)	14	1	
**Kumar (2022)** [34]	India	RCT	NR	Hamstring tendon	-	PRP	Tibial Tunnel	35	-	-	-	-	3 months
						Control	-	35	-	-	-	-	
**Azcárate (2014)** [25]	Spain	RCT	NR	Patellar tendon allograft	-	PRP	Tibial Tunnel	50	26.1	(14–57)	40	10	12 months
						Control	-	50	26.1	(15–59)	38	12	
**Vogrin (2010)** [53]	Slovenia	RCT	NR	Hamstring tendon	February–June 2008	PRP	Femoral and Tibial Tunnel	22	35.4	10	59.1	40.9	6 months
						Control	-	23	33	12.5	73.9	26.1	
**Gong (2022)** [31]	China	RCT	NCT04659447	Hamstring tendon	-	PRP	Femoral and Tibial Tunnel	30	33.5	8.97	18	12	12 months
						Control	-	30	34.9	9.68	21	9	
**Lin (2024)** [35]	Taiwan	RCT	NR	Hamstring tendon graft	-	Control	-	10	-	-	-	-	24 weeks
						PRP	Femoral Tunnel	8	-	-	-	-	
**Eslami (2022)** [29]	Iran	RCT	IRCT20200217046523N18	Not clarified	2020–2021	PRP	NR	50	32.26	3.69	-	-	10.26 (2.11) months
						Control	-	50			-	-	
**Ye (2024)** [56]	China	RCT	ChiCTR2000040262	Autologous semitendinosus and gracilis tendon	21 March 2021, 28 August 2023	PRP	Femoral and Tibial Tunnel	60	28	7.9	43	17	12 months
						Control	-	60	30	8	41	19	
**Walters (2018)** [54]	USA	RCT	NCT01765712	Patellar tendon	2011–2015	PRP	Not Clarified	27	30	12	-	-	24 months
						Control	-	23			-	-	
**Seijas (2013)** [43]	Spain	RCT	NR	Patellar tendon	January–July 2009	PRP	Femoral and Tibial Tunnel	49	-	-	-	-	12 months
						Control	-	49	-	-	-	-	
**Seijas (2015a)** [44]	Spain	RCT	NR	Patellar tendon graft	2009	PRGF	NR	23	20	3	-	-	4 months
						Control	-	20	17	3	-	-	
**Seijas (2015b)** [46]	Spain	RCT	NR	Patellar tendon	-	PRGF-Endoret	NR	50	-	-	-	-	12 months
						Control	-	50	-	-	-	-	
**Seijas (2016)** [45]	Spain	RCT	NR	Patellar tendon graft	2009	PRGF	NR	23	20	3	-	-	24 months
						Control	-	20	17	3	-	-	
**Vadalà (2013)** [51]	Italy	RCT	NR	Hamstring tendon	-	PRP	Femoral and Tibial Tunnel	20	34.5	(18–48)	20	0	14.7 months
						Control	-	20	34.5	(18–48)	20	0	
**Cervellin (2012)** [27]	Italy	RCT	NR	Patellar tendon	2008–2009	PRP	NR	20	22.9	4.3	20	0	12 months
						Control	-	20	22.7	3.5	20	0	
**Solomon (2022)** [48]	USA	RCT	NCT04993339	Peroneus longus tendon	2016, 2019	PRP	Femoral and Tibial Tunnel	13	32.7	10.5	8	5	6 weeks
						Control	-	14	32.7	12.7	7	7	
**Wasilczyk (2024)** [55]	Poland	RCT	NR	NR	March 2015, February 2024	Injected PRP	NR	30	46.5	15	16	14	6 weeks
						Control/PRP	-	10	33	12	9	1	
**Nin (2009)** [39]	Spain	RCT	NR	Patellar tendon	-	PRP	Tibial Tunnel	50	26.1	(14–57)	40	10	24 months
						Control	-	50	26.6	(15–59)	38	12	
**Silva (2009)** [47]	Portugal	RCT	NR	Hamstring tendon	November 2006–March 2008	PRP	Femoral Tunnel	30	-	-	-	-	3 months
						Control	-	10	-	-	-	-	
**Rupreht (2013)** [41]	Slovenia	RCT	NR	Semitendinosus and gracilis tendon graft	-	PRP	Tibial Tunnel	21	37.2	8.4	13	8	6 months
						Control	-	20	32.6	12.3	15	5	
**Vogrin (2010)** [52]	Croatia	RCT	NR	Hamstring tendon	February–October 2008	Control	-	20	32.6	12.3	15	5	12 weeks
						PG	Femoral and Tibial Tunnel	21	37.2	8.4	13	8	
**Rupreht (2013)** [42]	Slovenia	RCT	NR	Semitendinosus and gracilis tendon graft	-	PRP	Tibial Tunnel	21	37.2	8.4	13	8	6 months
						Control	-	20	-	-	15	5	
**Starantzis (2014)** [50]	Greece	RCT	NR	Hamstring tendon	December 2007–June 2010	PRP	Femoral Tunnel	25	29.4	7.3	38	13	12 months
						Control	-	26	31.3	8			
**Orrego (2008)** [40]	Chile	RCT	NR	Hamstring tendon	January 2005–December 2006	PC	Femoral Tunnel	26	-	-	-	-	6 months
						Control	-	27	-	-	-	-	
**Sözkesen (2018)** [49]	Turkey	RCT	Not registered	Hamstring tendon autograft	March 2014–July 2015	PRP	Femoral and Tibial Tunnel	18	26	6.96	16	2	12 months
						Control	-	26	26	6.96	25	1	
**Figueroa (2010)** [30]	Chile	RCT	NR	Hamstring tendon	-	APC	Femoral and Tibial Tunnel	30	26.8	(14–28)	18	12	6.4 months
						Control	-	20	23.6	(13–35)	15	5	
**Ji (2017)** [32]	China	RCT	NR	Hamstring tendon	August 2014–August 2016	PRP	NR	21	31.59	-	8	9	12 months
						Control	-	21	33.68	-	7	12	
**Kasl (2022)** [33]	Czech	RCT	NR	Hamstring tendon	2012–2014	PRP	NR	20	29.1	-	29	11	12 months
						Control	-	20		-			
**Zeman (2018)** [57]	Czech	RCT	NR	Semitendinosus and gracillis tendon	-	PRP	Femoral Tunnel	17	29.1	-	23	10	12 months
						Control	-	16		-			
**Munde (2023)** [38]	India	RCT	NR	NR	-	PRP	NR	40	-	-	-	-	6 months
						Control	-	40	-	-	-	-	
**Beyzadeoglu (2020)** [26]	Turkey	RCT	NR	Semitendinosus tendon graft		PRF	Femoral Tunnel	23	21.7		18	5	12 months
						Control	-	21	22.1		17	4	
**Malinowski (2021)** [36]	Poland	RCT	Not registered	Quadriceps tendon bone reconstruction	2008–2010	PRP	NR	54	-	-	-	-	18 months
						Control	-	52	-	-	-	-	

YOP: year of publication; YOI: year of investigation; RCT: randomized controlled trial; PRP: platelet-rich plasma; NR: not reported; SD: standard deviation.

**Table 2:** A summary of ligamentization between PRP and control groups based on MRI hypointensity grades.

	Hypointense			Grade 1 (Mildly Hypointense)			Grade 2 (Moderately Hypointense)			Grade 3 (Severely Hypointense)			Grade 4 (Diffusely Hypointense)		
	K	OR	95% CI	K	OR	95% CI	K	OR	95% CI	K	OR	95% CI	K	OR	95% CI
Risk of Bias															
High	3	0.03	0.01–0.11	3	0.31	0.14–0.68	3	0.93	0.38–2.27	3	1.09	0.65–1.82	3	2.61	0.80–8.52
Some concerns	4	0.09	0.02–0.58	3	0.29	0.13–0.64	3	0.95	0.40–2.24	3	1.06	0.64–1.74	3	2.72	0.82–9
Graft Type															
Hamstring tendon	1	0.63	0.21–1.93												
Patellar tendon	6	0.04	0.02–0.10	6	0.3	0.17–0.52	6	0.97	0.56–1.65	6	1.07	0.75–1.54	6	2.59	1.21–5.52
Injection Site															
Femoral and tibial tunnels	4	0.09	0.02–0.58	3	0.29	0.13–0.64	3	0.95	0.40–2.24	3	1.06	0.64–1.74	3	2.72	0.82–9
Not Clarified	3	0.03	0.01–0.11	3	0.31	0.14–0.68	3	0.93	0.38–2.27	3	1.09	0.65–1.82	3	2.61	0.80–8.52
Follow-up time															
4 months	2	0.06	0.02–0.19	2	0.32	0.16–0.66	2	1.67	0.94–2.97	2	1.41	0.71–2.79	2	1.14	0.73–1.78
6 months	3	0.08	0.01–0.86	2	0.19	0.19–0.58	2	0.59	0.30–1.13	2	1.07	0.59–1.94	2	3.24	1.02–10.30
12 months	2	0.01	0.00–0.30	2	0.5	0.09–2.79	2	0.5	0.09–2.79	2	0.86	0.47–1.58	2	7.33	2.99–17.99

OR: odds ratio; CI: confidence interval; K: number of studies included in each subgroup; MRI: magnetic resonance imaging.

## Data Availability

Data are available within the manuscript and its Supplementary Materials.

## Supplementary Materials

The following supporting information can be downloaded at: https://www.mdpi.com/article/10.3390/jcm15072526/s1, Supplementary File S1: a PRISMA checklist. Figure S1: a forest plot showing the difference in postoperative Lysholm score between PRP and control stratified by risk-of-bias level, graft type, injection site, and follow-up time; Figure S2: a Forest plot showing the difference in postoperative Tegner score between PRP and control stratified by risk-of-bias level, graft type, injection site, and follow-up time; Figure S3: a Forest plot showing the difference in graft maturation (SNQ score) between PRP and control stratified by risk-of-bias level, graft type, injection site, and follow-up time; Figure S4: a Forest plot showing the difference in postoperative femoral tunnel diameter between PRP and control stratified by risk-of-bias level, graft type, injection site, and follow-up time. Table S1: the detailed database search query employed in this systematic review.

## Abbreviations

The following abbreviations are used in this manuscript:

ACL	Anterior Cruciate Ligament
ACLR	Anterior Cruciate Ligament Reconstruction
AMSTAR	Assessing the Methodological Quality of Systematic Reviews
APC	Autologous Platelet Concentrate
BTB	Bone–Tendon–Bone
CI	Confidence Interval
IKDC	International Knee Documentation Committee
I^2^	Inconsistency Index
KT-1000	KT-1000 Arthrometer
MD	Mean Difference
MRI	Magnetic Resonance Imaging
NR	Not Reported
OR	Odds Ratio
PC	Platelet Concentrate
PDGF	Platelet-Derived Growth Factor
PICOS	Population, Intervention, Comparison, Outcomes, Study design
PRGF	Plasma Rich in Growth Factors
PRISMA	Preferred Reporting Items for Systematic Reviews and Meta-Analyses
PRP	Platelet-Rich Plasma
PRF	Platelet-Rich Fibrin
RCT	Randomized Controlled Trial
REML	Restricted Maximum Likelihood
SD	Standard Deviation
SNQ	Signal-to-Noise Quotient
TGF-β	Transforming Growth Factor Beta
VAS	Visual Analogue Scale
VEGF	Vascular Endothelial Growth Factor
YOI	Year of Investigation
YOP	Year of Publication
